# Semantic Annotation of Experimental Methods in Analytical Chemistry

**DOI:** 10.1021/acs.analchem.2c03565

**Published:** 2022-10-25

**Authors:** Magnus Palmblad, Enahoro Asein, Nina P. Bergman, Arina Ivanova, Lukas Ramasauskas, Hazzar Mohammed Reyes, Stefan Ruchti, Leonardo Soto-Jácome, Jonas Bergquist

**Affiliations:** † Center for Proteomics and Metabolomics, Leiden University Medical Center, 2300 RC Leiden, The Netherlands; ‡ Institute of Chemistry, University of Tartu, Ravila 14a, 50411 Tartu, Estonia; § Analytical Chemistry and Neurochemistry, Department of Chemistry—BMC, Uppsala University, SE-75124 Uppsala, Sweden; ∥ Analytical Pharmaceutical Chemistry, Department of Medicinal Chemistry - BMC, Uppsala University, SE-75123 Uppsala, Sweden

## Abstract

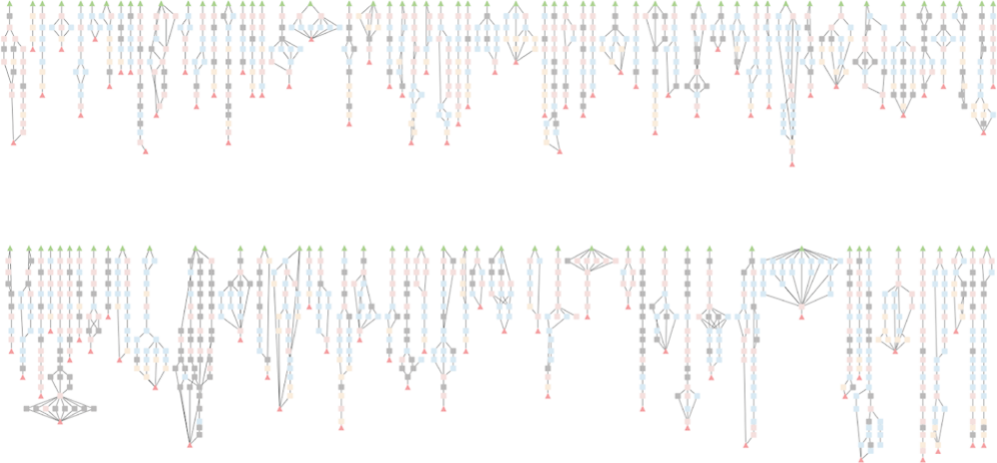

A major obstacle for reusing and integrating existing data is finding the data that is most relevant in a given context. The primary metadata resource is the scientific literature describing the experiments that produced the data. To stimulate the development of natural language processing methods for extracting this information from articles, we have manually annotated 100 recent open access publications in Analytical Chemistry as semantic graphs. We focused on articles mentioning mass spectrometry in their experimental sections, as we are particularly interested in the topic, which is also within the domain of several ontologies and controlled vocabularies. The resulting gold standard dataset is publicly available and directly applicable to validating automated methods for retrieving this metadata from the literature. In the process, we also made a number of observations on the structure and description of experiments and open access publication in this journal.

## Introduction

The scientific publication landscape is changing rapidly, with increasing emphasis on making the latest research available for everyone to read and reuse, aligning closely with the goals of FAIR^1^ movement. Open access, as first defined by the Budapest Open Access Initiative in 2002, gives everyone access to the latest scientific results and is a cornerstone of open science. Open access publications are not only “free to read” but also “free to use” for text mining and integrating data across publications. Open access enables computational analysis of full text articles, retrieving information from specific sections, such as the methods or results, and reusing this information.

The primary problem in large-scale data integration is finding the relevant data in the first place. This often requires searching for the associated metadata in data repositories or the scientific literature directly. Though many repositories provide formalized metadata using controlled vocabularies (CVs) or ontologies, the information is often incomplete, referring to a published article for the details. Provided these articles are open access, we can search for them automatically. Of course, the articles are written in natural language, requiring natural language processing (NLP) to progress beyond simple matching of words. One of the main obstacles for widespread application of NLP is the lack of appropriate training data, such as a large annotated corpus of scientific articles. Recently, transfer learning methods for NLP such as BERT^2^ have been used to apply previously trained models on different corpora with significant success. Text mining has also been used to mine the scientific literature for bioinformatics software,^3^ including matching the descriptions of the software to an existing ontology, EDAM,^4^ and build entries for the bio.tools software registry.^5^ However, many challenges remain in applying and evaluating NLP and text mining methods in new areas, such as finding interoperable data that was acquired using similar (or complementary) analytical methods. To stimulate the development of NLP models in the field of analytical chemistry and provide a gold standard reference set for validation of such methods, we set out to semantically annotate 100 experimental sections describing analytical chemistry experiments from the samples to the statistical analyses and data presentations.

A major inspiration for this work is the SMART Protocols by Giraldo and co-workers,^6^ proposing a semantic representation for experimental protocols in the biomedical literature. Rather than trying to capture all information on samples, instruments, reagents, and research objectives, as in the SMART Protocols model, we take a simpler approach and focus on capturing those key steps of the method that are either transformative or generative, that is, materially change a sample, generate information on the sample (by an analytical procedure such as mass spectrometry), or transform the data from one form into another. However, for these key steps, we aim to be as specific as possible to capture critical metadata that would be needed in data integration. We are here less concerned with the specific samples analyzed or the research objectives, but like Giraldo et al. we also aim to create a set of expert curated experimental methods for validation of automated methods for retrieving the same information. Where Giraldo et al. cast a wide net and collected protocols on different topics from 10 repositories and journals, we focus on methods involving mass spectrometry and recently published in Analytical Chemistry. We deliberately choose research articles rather than protocol publications, as the former are typically referenced by the repositories.

More recently, but also noteworthily, Brown et al.^7^ described the much larger effort of the RELISH (RElevant LIterature SearcH) consortium to curate over 180,000 articles with respect to relevance (similarity) to a seed article and made the results available as a resource for testing and improving biomedical literature recommender systems. This addresses a more general problem of finding the most relevant literature, which may also be helpful for finding relevant data for comparison with a given dataset.

In addition to creating this gold standard annotated corpus of experimental sections, we also report observations on the structure of experimental sections, how they are written, how mass spectrometry is combined with other analytical techniques, and on open access publication in analytical chemistry in general, as they too may be of interest to the readers of this journal.

## Methods

### Curators

The main curation effort was conducted during a January 2022 Winter School by 35 first- and second-year students in the Erasmus Mundus “Excellence in Analytical Chemistry” (EACH) international master’s programme at the University of Tartu, Estonia, Åbo Akademi University, Finland; Uppsala University, Sweden; and University Claude Bernard Lyon 1, France. All students have similar and strong backgrounds in analytical chemistry and metrology, and specialize in topics such as organic and bio-organic analysis, advanced separation methods, mass spectrometry, electrochemistry, sensors, miniaturization, industrial analysis, and process control. During the initial curation phase, the students worked in teams of three or four.

### Corpus

The corpus was defined as all “free to read and free to use” open access articles published in Analytical Chemistry in 2020, 2021, or the first six months of 2022 explicitly mentioning mass spectrometry in their experimental section. These articles were retrieved using the Europe PMC^8^ search query: “(JOURNAL:”Analytical Chemistry”) AND [FIRST_PDATE:(2020-01-01 TO 2022-06-30)] AND (METHODS:”mass spectrometry” OR METHODS:”MS” OR METHODS:”mass spectrometer”) AND (OPEN_ACCESS:y)” returning 220 publications when executed on August 15, 2022, 72 from 2020, 76 from 2021 and 72 from the first half of 2022. The query was executed and the article metadata and full text XML downloaded in R using the europepmc package version 0.4.1. Corresponding author affiliations were analyzed to determine any bias with respect to the country of the corresponding author. The experimental section was extracted using XPath, and the word count was calculated using the ngram package version 3.2.0. The curators worked from the PDF version of the articles.

### Method Annotation

Methods were annotated as semantic directed acyclic graphs (DAGs) in the Graph Modelling Language (GML),^9^ with nodes representing the transformative or generative steps of the methods as identified by the curator, for example, those steps that substantially change samples, analytes or data (all represented by edges), or generate data from a sample. Directed graphs were chosen as they explicitly describe the order of the steps in the method. This order is critical—for example, different orders of chemical derivatization or whether proteins are digested before or after a chromatographic separation are different experiments yielding different information. The GML format was chosen as it is a simple, human- and machine-readable format that the curators could easily understand and work with, and that can be read by many applications, including Cytoscape^10^ and igraph.^11^ The GML files can also be converted to RDF using igraph.

**Figure 1:**
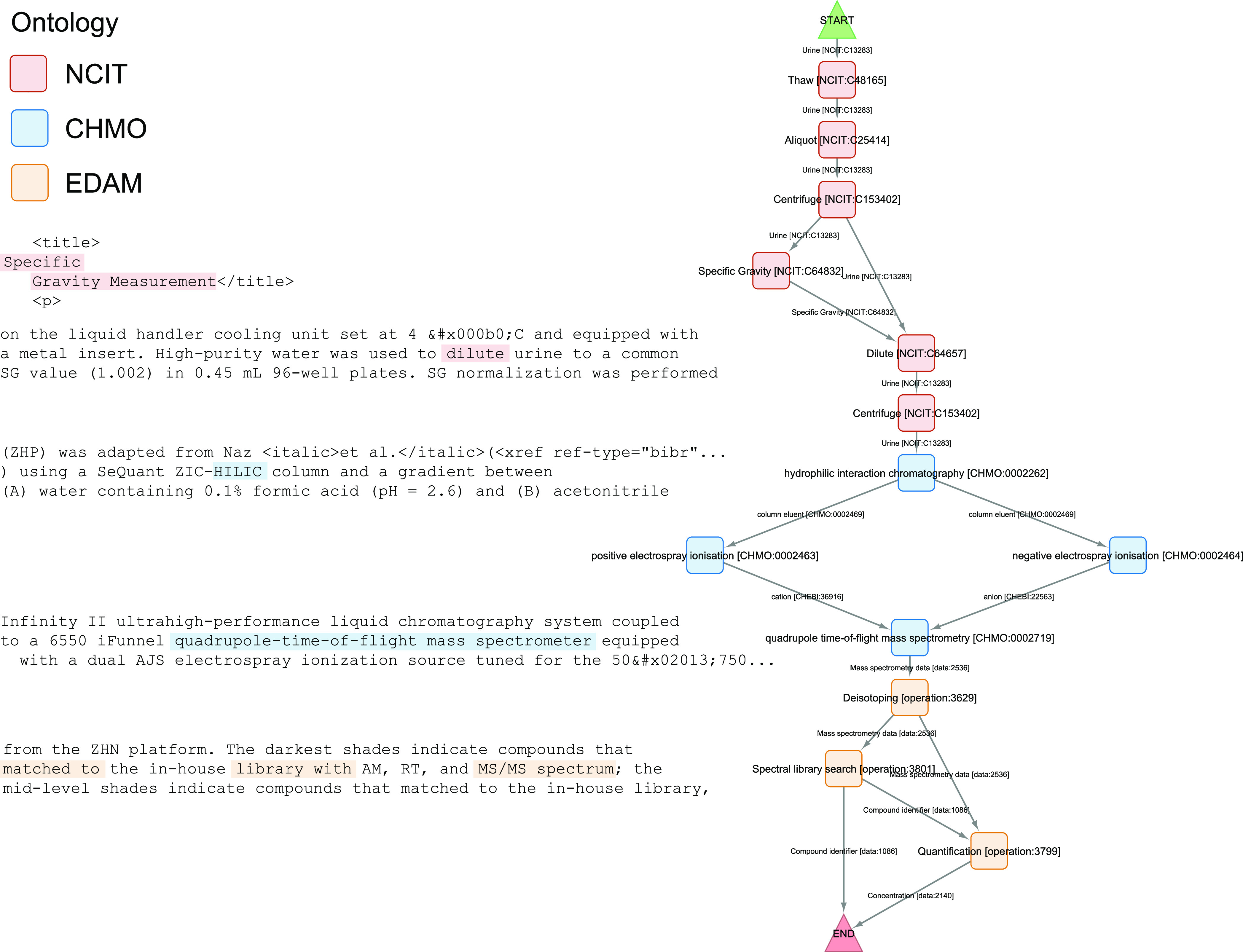
Example annotation of Meister et al.^15^ provided to the curators before the exercise, with the minimum information required to annotate four of the nodes highlighted in the fulltext XML excerpts. The information is neither contiguous nor given in the same order as in the actual experiment. This article was selected as an example, as it already had a similar graph-based description of the experimental workflow in its Figure 1. Other examples in the corpus are Ross et al.,^16^ Yan et al.,^17^ Evers et al.,^18^ van Mourik et al.,^19^ and Graça et al.^20^ The majority of papers have a simplified schematic representation of the experiment or study as their table of contents/abstract graphic. The full text XML is readily available for all articles in the corpus and can be downloaded automatically from Europe PMC without a subscription to or special agreement with the journal. The graph was rendered in Cytoscape using the yFiles^21^ hierarchic layout.

The curators were instructed to construct a DAG representing the method described in the experimental section of each paper, labeling the nodes and edges using any ontology included in the Ontology Lookup Service^12^ (OLS). Curators were asked to select the term that most closely represents the identified steps in the method regardless of ontology, but with preference given to three ontologies, NCIT,^13^ CHMO,^14^ and EDAM,^4^ for terms included in more than one ontology. The curators were shown an example (Figure 1) and instructed to use action nouns, for example, “mass spectrometry” (CHMO:0000470) rather than “mass spectrometer” (CHMO:0000982). The curators were also instructed to be as precise as possible, for example, using the “positive electrospray ionization” (CHMO:0002463) or “negative electrospray ionization” (CHMO:0002464) terms when the ion source polarity is given, and both terms if polarity was switched during the experiment (as in that described in Figure 1), rather than the parent term “electrospray ionisation” (CHMO:0001659). Strictly speaking, NCIT is not an ontology but a reference terminology. However, it is available in the OBO (Open Biomedical Ontologies) format and can be browsed in the same way as the true ontologies. In this work, we did not perform any logical reasoning over the ontologies or attempt to merge them. The same applies to PSI-MS and other CVs searched by the OLS, which were also used in the annotations when a matching term could not be found in the preferred ontologies. In addition to labeling the nodes and edges using both the class label (e.g., “electrospray ionization”) and ID (CHMO:0001659) from the ontologies, curators were instructed to make notes of problematic, partial or imprecise annotations, or when critical steps were inferred from context using expert knowledge and experience. These notes are provided as comments in the GML. All annotations were checked by at least one other curator. To study the concordance of the annotations, three articles selected at random were annotated independently by five curators.

To make the GML files compatible with Cytoscape, node labels were made globally unique by concatenating the article ID from its DOI with the node number in each graph using R 4.1.2 with igraph 1.2.7. Cytoscape version 3.9.1 was used to visualize, inspect, and merge annotations, with the loading and visual style controlled from R using RCy3 version 3.14. All R scripts, GML files, and resulting Cytoscape session files are available on GitHub (https://github.com/magnuspalmblad/EACH). Review papers, comparisons of large numbers of methods, and interlaboratory studies were excluded from the annotation, as they generally do not describe primary methods in detail.

## Results and Discussion

### General Observations of the Corpus

**Figure 2:**
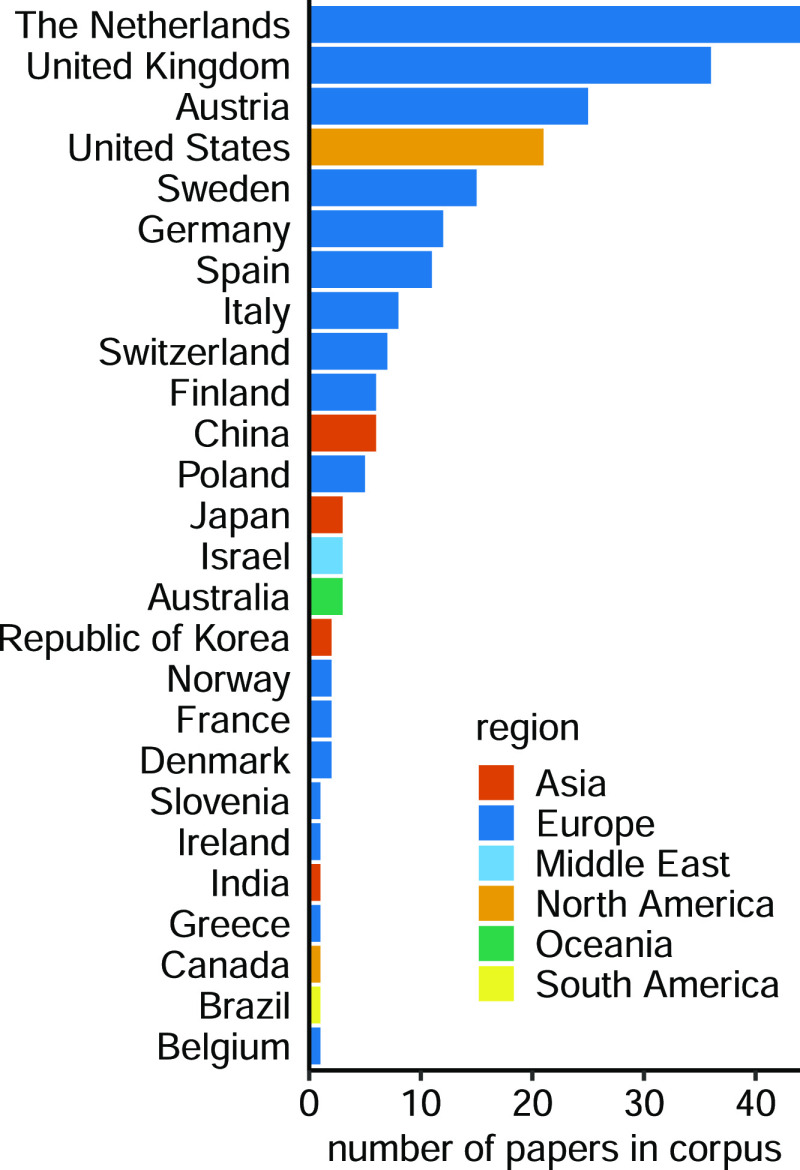
Corresponding author affiliation by country, showing the dominance of European authors (81.3%) in this corpus. This is likely a consequence of funding agreements for open access publishing between ACS and partnering universities in countries like the Netherlands and Austria that remove the direct financial burden from the authors.

The distribution of the corresponding author countries (Figure 2) shows that European corresponding authors, in particular those from the Netherlands (20%, or 44 out of 220 articles) and Austria (10.5%, 23/220 articles), are overrepresented in the corpus. Proportionately, authors from the United States and China are clearly underrepresented with only 21 and 6 articles, respectively, whereas nearly half (47.3% or 203/426 articles) of the articles matching the search query without the open access requirement are from the United States. Full-text deposition in PubMed Central is common in the US, but these articles are not generally open access, as in “free to read and free to use,” and therefore not automatically included in the corpus. The reverse is true; however, all articles in the corpus are also in PubMed Central. When expanding the analysis to look at all 5100 articles in Analytical Chemistry published in 2020, 2021, or before July 1, 2022, we see that the plurality of corresponding authors (45.9% or 2343 articles) are from China, with 20.0% (1019 articles) from the United States. The reason for the dominance of European authors in our corpus is likely a combination of a number of productive research groups with a long-standing interest in mass spectrometry and a more recent emphasis on open access publishing, including institutionally sponsored open access agreements with the publisher in several countries, such as the Netherlands and Austria. Researchers mining the open access literature should be aware of potential bias introduced by this significantly varying commitment to open access between countries and the consequently varying coverage of the research output from different countries in the open access literature. Our corpus covers 100% of the output from the Netherlands and Austria but only 10.4% of the output from the United States.

### Ontologies

Several general ontologies have been developed with the aim to cover methods in the biomedical and biological sciences. However, the curators found quickly that no single ontology domain covers all important aspects of methods, even in this narrowly defined corpus with a single topic (mass spectrometry) in a single journal (Analytical Chemistry). Of all the ontologies and thesauri in OLS, the NCIT reference terminology possibly comes closest, with its 172,472 terms (April 20, 2022 version) providing coverage ranging from describing the sampling biological systems to sample preparation, analytical chemistry, and data analysis operations. However, NCIT is designed for the cancer domain, including related diseases and research findings. This means the connotation most common within that domain takes precedence. For example, infusion, or “Infusion Procedure” (NCIT:C15388) is defined as “Any form of treatment that is introduced into the body via a blood vessel, a muscle, or the spinal cord.” Although the syringe pumps may be similar, infusion of a liquid sample into a mass spectrometer as in Camperi et al.^22^ is clearly better described by the term “infusion” (MS:1000060), defined as “The continuous flow of solution of a sample into the ionization source”. There are many homonyms in different ontologies and sometimes even words in one ontology can have different meanings depending on the context—for example, “alignment” in EDAM refers to either alignment of (discrete) sequences or (continuous) chromatograms, and “embedding”, which is used to describe the physical surroundings of an object as in “paraffin embedding” as well as a statistical transformation of data as in “t-distributed stochastic neighbor embedding”. During the annotations, some lacunae were identified and communicated to the maintainer of CHMO, resulting in the addition of terms such as the action noun ”orbitrap mass spectrometry” (CHMO:0002926) and a new class “Fourier transform mass spectrometry” (CHMO:0002925) as the parent to both “orbitrap mass spectrometry” and “Fourier transform ion cyclotron resonance mass spectrometry” (CHMO:0000502). Previous annotations were revised with these new terms.

**Figure 3:**
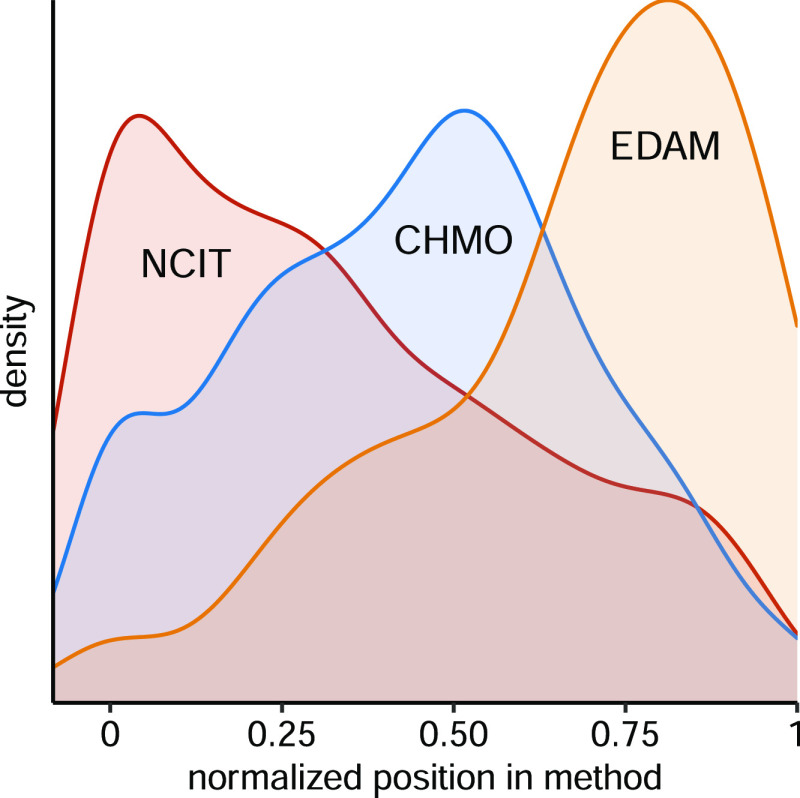
Ontology or reference terminology as a function of normalized distance from the start node in the 100 annotated methods, showing kernel density estimates of the distribution from the closest (0) to the furthest (1) annotated step. The less frequently used controlled vocabulary PSI-MS has a mean of 0.53, between CHMO at 0.43 and EDAM at 0.68. The statistical ontology STATO has a mean of 0.77, closest to the end of methods.

Most method descriptions in experimental sections start by describing the samples or analyzed materials, before proceeding with the sample preparation and analysis. We found that general ontologies and thesauri such as NCIT cover the first general steps quite well, and CHMO takes over when more precise analytical chemistry terms are needed. Finally, EDAM has a fairly rich set of terms to describe data analysis operations, especially involving mass spectrometry and proteomics. Figure 3 shows how these three ontologies cover different phases of experimental methods, from the sample to final data analysis. There are some interesting exceptions, however, such as Meekel et al.,^23^ which begins with an advanced computational analysis, predicting toxicity of compounds, identifying structural patterns, and calculating theoretical mass spectral features from these patterns. The actual measurements validating this computational method are described in relatively little detail, as the computational method is the major emphasis of the paper. Consequently, the terms most precisely matching those in the first part of the method description were found in EDAM rather than in NCIT or CHMO.

### Relationship to Original Text

**Figure 4:**
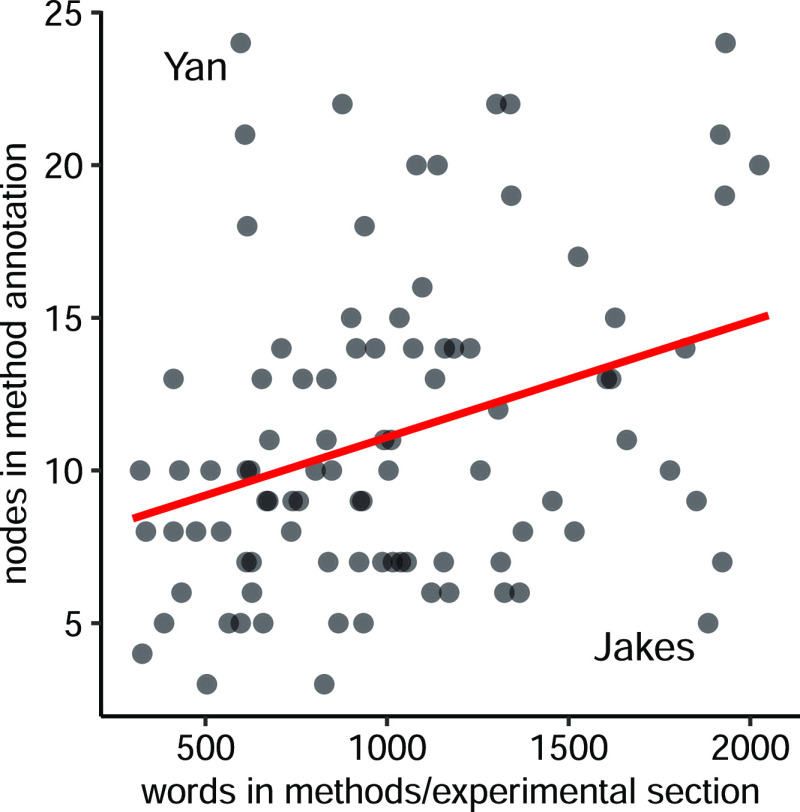
Number of nodes in semantic method annotations as a function of word count in the corresponding experimental section, with the two discussed examples of Yan et al.^17^ and Jakes et al.^25^ indicated. The red line represents the linear regression, *R*^2^ = 0.1912, *p* = 6.0 × 10^–6^, that is, a weak but statistically significant correlation between the word count and the number of nodes. One annotated article^26^ with the methods provided as Supporting Information was excluded from the analysis.

The length of the average experimental section in the corpus is 1078 words, slightly longer than the 978 word average for all 447 open access articles in nalytical chemistry published in the same time period but considerably shorter than the average in the Journal of Proteome Research of 1377 words, suggesting that proteomic experiments are relatively more complicated, or at least require more words to describe. The correlation between the number of curated key steps described in the experimental section and the word count in the section is surprisingly poor (Figure 4). In part, this may be explained by the varying use of compound terms between annotations. Some ontologies, in particular CHMO, are rich in compound concepts that correspond to hyphenated methods in analytical chemistry. For example, one experiment described in Schoeberl et al.^24^ was annotated with the single term “laser ablation inductively coupled plasma time-of-flight mass spectrometry” or LA-ICP-TOFMS (CHMO:0000551). But it could just as well be annotated by connecting the individual terms “laser ablation” (CHMO:0001132), “plasma ionization” (CHMO:0001665), and “time-of-flight mass spectrometry” (CHMO:0000580), in that order. Similarly, a common method in proteomics experiments could be annotated with the compound “reversed-phase liquid chromatography-electrospray ionization tandem mass spectrometry” (CHMO:0000738), or by three or more primitive terms. The compound terms simplify annotation, but the primitives are needed to describe new methods or variants of existing methods. However, most of the variability comes from the different writing styles of authors, where some take a minimalist approach to describing their methods and others, such as Jakes et al.,^25^ provide theirs in prose that is simultaneously clear and verbose. The opposite extreme in Figure 4 is Yan et al.^17^ comparing three feature selection methods and eight machine learning algorithms. These are all listed in the methods section with citations to other work describing them in detail, in what is accepted practice.

### Method Graphs

**Figure 5:**
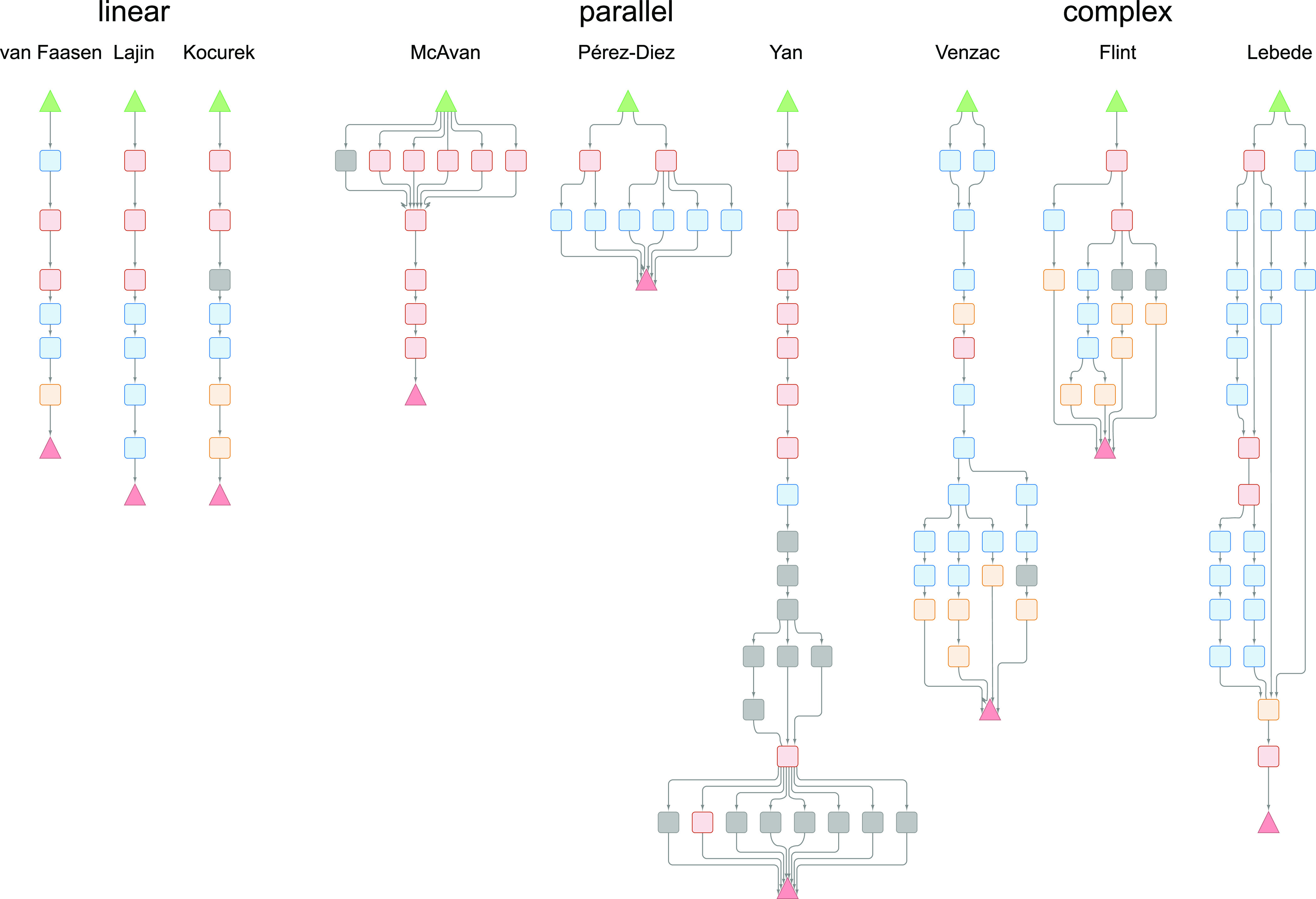
Graph representations of the linear experimental sections in van Faassen et al.,^28^ Lajin and Goessler,^29^ and Kocurek et al.^30^ (left), ”parallel” experiments in McAvan et al.,^31^ Pérez-Diez et al.,^32^ and Yan et al.^17^ (middle), and more complex methods in Venzac et al.,^33^ Flint et al.,^34^ and Lebede et al.^35^ (right). The graphs were rendered and the nodes colored as in Figure 1. The full annotation details are available in the Cytoscape file on GitHub.

In total, we annotated 100 experimental sections as method graphs with a total of 2701 semantic annotations—1185 nodes (excluding START and END nodes) and 1516 edges, that is averaging to around 12 nodes and 15 edges per graph. Of these methods, 27 are annotated as linear DAGs, with all nodes (except the terminal nodes) having a degree of 2, whereas a few others have many parallel branches (two nodes of degree ≫2) or exhibit a more complex branching pattern, with many nodes of degree >2. Figure 5 shows three annotations of each type. The full details with node and edge labels are available in the Cytoscape file on GitHub. Capturing this experimental structure is relatively easy for human experts but a challenging task for computers, especially if without access to figures illustrating the experimental workflow. The types and their delineation are somewhat arbitrary but could be assigned from node degree distributions (Supporting Information Figure S1). Unsurprisingly, the concordance between curators was not perfect. Annotations made by different experts differed primarily in the level of detail, with some using more terms to describe the same step of the experimental method. Some papers, for example, Molenaar et al.,^27^ contained significant parts of the described method in the results section. These parts were not annotated here.

Looking deeper at the annotations, we see that liquid chromatography is by far the most common separation technique, with direct infusion, electrophoresis and direct tissue sampling in MALDI–IMS or LESA also appearing in the annotated experimental sections. However, it is often hard to delineate precisely which word or phrase determines the annotation. Frequently, clues to an annotation are scattered throughout the methods section. Often, there are redundancies, and one word or phrase out of several would suffice. Sometimes, key information is inside a figure or table of content graphics depicting the experimental workflow. To our knowledge, there is currently no markup or annotation software or standard that allows the definition of such complex relationships between the text (and figures) and matching concepts in an interactive and user-friendly manner.

The node-centric view emphasizes the general aspects of the experiment rather than the specific samples that were used to demonstrate the method. The edges, especially between the first few nodes, typically refer to specific cell lines, proteins, and other analytes. Edges between downstream nodes tend to receive more general annotations, such as “peptide,” “ion,” “mass spectrum,” “concentration” or “chromatogram visualization”.

The EACH annotations are high-level, abstract method representations without text tagging (except for the examples above). They may be useful in evaluating the overall accuracy of trained NLP models but not which words or phrases are recognized or how they are combined. Clues are distributed throughout the experimental section, often also in figures and repeated in other sections of the article. Some information can only be inferred from instances rather than recognition of entities (classes) in an ontology. For example, from reading “Sephadex” and knowing this is a gel filtration medium, we infer that gel filtration chromatography (CHMO:0001011) was conducted, even when there is no mention of gel filtration anywhere in the article.^36^ Even in the much more limited namespace of commercially available mass spectrometers, there is no ontology that links instrument models with instrument types. The PSI-MS controlled vocabulary currently (2022-08-15) includes 56 commercially available and named “Thermo Scientific instrument model” models in the “instrument model” branch, but does not specify any relationships between these and mass analyzer types in the “mass analyzer” branch. These would arguably be easy to add and far more informative on the dimensionality and quality of the data than knowing only the manufacturer, even though file formats and software compatibility often do depend on the latter. However, the PSI-MS is a controlled vocabulary and not an ontology and was not designed with the goal of making these types of inferences possible.

While more limited in scope than efforts such as SMART Protocols and RELISH, the 100 EACH annotations may be able to stimulate work on comparing experimental designs, particularly in analytical chemistry, between research publications or data repositories. Such metrics would be of particular relevance in large-scale automated data integration efforts, including querying the primary literature for data suitable for comparison with a given (seed) dataset. The EACH annotations may provide answers to some basic questions important in evaluating automated methods mining method descriptions, including if all key steps are recognized, that the order of steps is correctly inferred, and that any branches are correctly identified. As the curators were free to use any ontology and worked independently or in small groups, the exercise also revealed which ontology, among those in the OLS, is most fit-for-purpose for annotating a given type or stage of method. The range of terms and indeed the number of ontologies and reference terminologies required to precisely annotate the experimental and data analysis methods combined with mass spectrometry underscores just how versatile mass spectrometry is.

Future efforts may include mapping all annotations to a single ontology and add text (XML) markup, qualifying each annotation with what words or phrases were used to infer the annotation, in which combination, and whether the information was necessary or sufficient. Specific suggestions for improvements to individual annotations are welcome as issues or pull requests on GitHub (https://github.com/magnuspalmblad/EACH). Annotations of other papers within the corpus or older “free to read and free to use” open access papers in the same journal are equally welcome. There are currently (2022-08-15) 1051 such articles published in Analytical Chemistry, starting in 2008, approximately half (528) of which explicitly mention mass spectrometry in their experimental sections. As we have already annotated 100 or 19% of these with a small group of curators, it is entirely feasible to annotate most of the open access articles on mass spectrometry and even to broaden the scope to eventually reach one thousand semantically annotated experimental sections in open access Analytical Chemistry papers.

## Supporting Information Available

Node degree statistics (PDF)
