# Supplementary material for: Semantic Annotation of Experimental Methods in Analytical Chemistry
Source: Anal Chem. 2022 Oct 25;94(44):15464–71. doi: 10.1021/acs.analchem.2c03565 (PMC9647698; doi:10.1021/acs.analchem.2c03565)
Supplement: Supplementary file 1 — ac2c03565_si_001.pdf [file ac2c03565_si_001.pdf]

Supporting Information
Semantic Annotation of Experimental Methods in *Analytical Chemistry*

Magnus Palmblad,[*,†] Enahoro Asein,[‡] Nina P. Bergman,[¶] Arina Ivanova,[¶] Lukas Ramasauskas,[¶] Hazzar Mohammed Reyes,[‡] Stefan Ruchti,[‡] Leonardo Soto-Jácome,[‡] and Jonas Bergquist[¶]

†*Center for Proteomics and Metabolomics, Leiden University Medical Center, 2300 RC Leiden, The Netherlands*

‡*Institute of Chemistry, University of Tartu, Ravila 14a, Tartu, 50411, Estonia*

¶*Analytical Chemistry and Neurochemistry, Department of Chemistry - BMC, Uppsala University, SE-75124 Uppsala, Sweden*

E-mail: n.m.palmblad@lumc.nl

Phone: +31 71 5266969

Table of Content

1. Node degree statistics for methods labeled as linear, parallel and complex, Figure S1

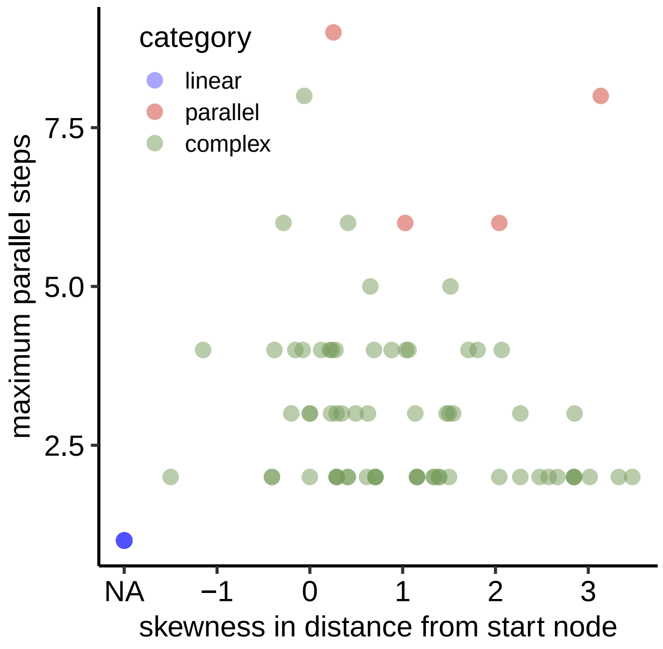

Figure S1: Maximum number of parallel nodes as function of the skewness of node degree distributions for linear, parallel and complex methods. Perfectly linear methods have at most one parallel node, and therefore undefined skewness. The parallel methods have a large maximum number of parallel nodes and high node degree distribution skewness, even if the border between what is considered parallel and complex is somewhat arbitrary.
